# Hydrogel-Based Semiconductors: Principles, Types, and Emerging Applications

**DOI:** 10.3390/gels12050419

**Published:** 2026-05-11

**Authors:** Md Murshed Bhuyan, Kyungjun Lee, Jae-Ho Jeong

**Affiliations:** 1 Department of Mechanical, Smart, and Industrial Engineering (Mechanical Engineering Major), Gachon University, 1342 Seongnam-daero, Sujeong-gu, Seongnam-si 13120, Republic of Korea; mdmurshed86@gachon.ac.kr; 2 School of Mechanical Engineering, College of Engineering, Chung-Ang University, 84 Heukseok-ro, Dongjak-gu, Seoul 06974, Republic of Korea

**Keywords:** gels/hydrogel, semiconductor, electronics, biocompatible, doping

## Abstract

The world’s current technical developments are mostly dependent on semiconductors. Even though traditional semiconductor materials are important, they have various disadvantages, especially when evaluated against polymer-based alternatives. Hydrogel-based semiconductors provide soft, ionically linked electronic interfaces by combining hydrated, mechanically compliant matrices with electrically active conjugated polymers and composites which can be applied in bioelectronic and thermoelectric generator/cells. Volumetric capacitances are normally in the range of 1–485 F·cm^−3^, demonstrating excellent ion storage, transport capabilities, and electron mobilities for hydrogel semiconductors spanning roughly 0.25 cm^2^/V·s (measured for n-type P(PyV)-H hydrogel). The fabrication techniques include additive free casting and room-temperature crosslinking, which lower energy input while maintaining electronic performance; typical systems maintain >80% of their conductivity after $10^{3}$–$10^{4}$ mechanical cycles. This review study mainly focuses on the design, preparation, application, and prospects of gel/hydrogel-based semiconductors. It gives readers a thorough understanding of the basic ideas that underline their structure and operation. All things considered, this work is a useful tool for engineers and researchers looking to maximize the potential of gel-based semiconductors in next-generation electrical systems.

## 1. Introduction

Hydrogels are soft, tissue-like networks of hydrophilic (water-loving) polymer chains that have the capacity to absorb and retain vast quantities of water [1]. Semiconductors are essential components for electronics because of their perfectly controllable electrical conductivity [2]. An organic semiconductor is a material whose electrical conductivity lies between that of conductors and insulators, defined by distinct ‘energy levels’ formed by molecule orbitals, in contrast to the continuous energy bands seen in inorganic semiconductors. Specifically, the energy levels of the highest occupied molecular orbital (HOMO) and the lowest unoccupied molecular orbital overlaps, as well as the energy band gap between them, are closely related to π-bonding orbitals and quantum mechanical wave-function overlap, which are essential for charge transport in these materials. Because of their potential uses in various electronic devices, including organic field-effect transistors (OFETs), organic light-emitting diodes (OLEDs), and organic photovoltaic cells (OPVs), organic semiconductors have attracted a lot of attention. Molecular design can be used to adjust their conductivity. Conventional inorganic semiconductors have delocalized charge carriers due to their continuous energy bands. On the other hand, charge transport in organic semiconductors relies on carriers hopping across molecules and has defined energy levels (molecular orbitals). Organic materials can have special qualities because of this basic distinction. The intrinsic flexibility and low temperature processability of organic semiconductors make them noteworthy. Traditional inorganic semiconductors, on the other hand, are usually stiff and frequently need high-temperature processing, which restricts their use in flexible or conformable electronics [3]. Carbon-based materials known as organic semiconductors have a number of difficulties, including low thermal stability, grain boundaries and morphological flaws, short operating lifespan, and environmental instability. Semiconductor polymers are thought to be better than inorganic semiconductor materials like silicon nanostructures or quantum dots because they are more biocompatible and biodegradable. This lessens worries about the toxicity that heavy metal ions might cause in living things, which makes them better suited for in vivo applications [4,5]. Semiconducting polymers have high light-absorption coefficients and optical characteristics that may be precisely adjusted [6]. Colloidal nanoparticles with regulated sizes and shapes may be produced by processing semiconductor polymers, which is an improvement over materials like polydopamine nanoparticles (PDA NPs), which frequently suffer from uncontrolled particle sizes.

Gel-based semiconductors are a type of organic semiconductors with special qualities, including stretchability, self-healing, biocompatibility, electrical conductivity, and stimulus responsiveness. They are being developed to lessen the drawbacks of organic and inorganic semiconductors. A hydrogel-based semiconductor incorporates semiconducting molecules or polymers into the hydrogel matrix [7,8]. Gel-based semiconductors have the potential to be very important in flexible electronics, sophisticated optoelectronics, and other smart devices because of their adjustable characteristics and versatility in numerous material forms [9,10]. Hydrogel-based semiconductors, notably those that use semiconducting polymers like PDPPEDOT, overcome the drawbacks of earlier technologies and provide a number of benefits for biomedical applications, particularly in drug delivery and controlled release. Their sensitive behaviors and special material qualities are the source of these advantages. The hydrogels made from semiconducting polymers exhibit quick and reversible mechanical shrinking when exposed to light. In order to overcome the drawbacks of conventional hydrogels with their delayed switching kinetics, this quick and reversible volume shift is essential for pulsatile drug release and other biological applications. Yingjie Wu et al. prepared a near-infrared (NIR) photothermal-responsive polymer composite hydrogel by combining N-isopropylacrylamide with a narrow-bandgap semiconductor polymer. When exposed to NIR light, these hydrogels show a quick and reversible mechanical shrinkage. They can be utilized to load anticancer medications and regulate the release of such chemicals in space and time. Biocompatibility, high compressibility, water content, reversible volume change, and adjustable qualities are the main reasons why hydrogels are valued [11]. Even though gel-based semiconductors have problems with grain boundaries and their intrinsic insulating properties, metal ion doping and creative manufacturing methods like thin films and mixed-matrix membranes have led to notable breakthroughs. Their wider application in flexible and responsive optoelectronic systems is made possible by these advancements in conductivity, mechanical characteristics, and optical tunability [12]. There are drawbacks to gel-based semiconductors as well, namely their slower electrical reaction and difficult miniaturization. However, in applications such as bioelectronics or flexible sensors, they frequently perform better in terms of robustness and environmental tolerance than organic semiconductors. This overview explains how hydrogel works as a semiconductor, the state of research, and the potential for creating better gel-based semiconductors in the future.

## 2. Hydrogel Semiconductors

Advanced bioelectronic applications can take advantage of hydrogels, which are often recognized for their ionic conductivity, by engineering them to have semiconducting characteristics. For dual electronic/ionic conductivity, conductive micro- and nanocomposites or ionic species are used. Semiconducting hydrogels are thought to be appropriate bio-interfacing materials for bioelectronic signal amplification and active filtering. They can alter and improve input signals by acting as channel materials in transistors, allowing for switching, amplification, rectification, and other logic functions. In contrast to conventional organic semiconductors, they are able to capture and amplify electrophysiological signals on site, lowering cable noise and increasing signal-to-noise ratios (SNRs) while maintaining strong signal amplification capabilities. For example, SNRs are greatly increased, and EOG and ECG signals recorded by these flexible amplifiers can be much higher than those obtained with standard gel electrodes [13,14]. Hydrogel semiconductors function by a mixed conduction mechanism that includes both electron and ion transport, especially when it comes to organic electrochemical transistors (OECTs). Their ability to convert biological impulses into electrical ones allows for applications like lactate or glucose concentration monitoring [15].

In an organic semiconductor (OSC) material, electrons are carried down the conjugated polymer backbone, while ions travel through the majority of the substance. This ionic migration is essential for controlling the OECT’s current. The n-type organic semiconductor accumulates charge carriers as a result of positive ions from the aqueous electrolyte migrating into it when a positive gate bias is applied [16]. The role of swelling and material properties is vital in affecting the working mechanism of hydrogel semiconductors. The OSC film swells when water molecules and ions migrate into it. The OSC’s morphology and chemical makeup determine this swelling. Swelling is advantageous for effective ion absorption at low operating voltages, but too much swelling might impair mobility by limiting charge transport routes and upsetting the microstructure’s crystallinity [17,18]. One common method for promoting both electrical and ionic transport is the use of oligo (ethylene glycol) (OEG) sidechains. During electrochemical doping, these glycol chains’ intrinsic polarity improves ion penetration into the polymer bulk [16]. Glycol sidechains, which are frequently used to make materials hydrophilic, encourage aqueous swelling and subsequent ion transport into the active layer, which raises the volumetric capacitance [18].

**Figure 1:**
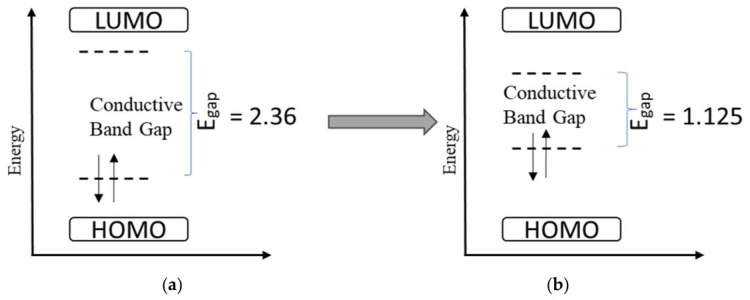
Conductive band gap energy: (**a**) silk fibroin composite hydrogel and (**b**) after PEDOT: PSS fabrication on silk fibroin [22].

Both electronic and optoelectronic functions, such as charge transport modulation, amplification, photoexcitation, photoluminescence, and photocatalysis, are demonstrated by the hydrogel semiconductors. Thus, it is quite desirable to give hydrogels semiconducting qualities. However, the charge carrier mobility of the described hydrogel semiconductors is now limited to transistors and is only 0.25 cm^2^ V^−1^·s^−1^. A polymer semiconductor with hydrophilic side chains and a hydrogel-forming polymer are the two primary components of Yahao Dai et al.’s hydrogel semiconductors with an interpenetrated network shape. They employed acrylic acid (AAc) as the hydrogel-forming monomer due to its strong reactivity in DMSO, and poly (3,3′-bis(2-(2-(2-methoxyethoxy)ethoxy)ethoxy)2,2′:5′,2′-terthiophene) p(g2T-T) as the polymer semiconductor. The crosslinking density of the PAAc network and the weight ratio between p(g2T-T) and PAAc are the two primary compositional parameters that can affect both electrical and mechanical capabilities. The hydrogels’ porous structure makes it more effective for redox processes to use electrons and holes [19]. Usually, the band gap energy of semiconductors is <3 eV. Depending on the kind of doping, the band gap of hydrogel semiconductors is thought to range between 1 and 2.5 eV [20]. Because it is mostly dependent on the particular conductive fillers (such as PEDOT: PSS, carbon nanotubes, or MXene) and the polymer matrix utilized, the band gap energy of conductive hydrogels is not a single constant number. In general, the pure, insulating hydrogel becomes a smaller-bandgap semiconductor instead of a wide-bandgap insulator when conductive elements are added. For instance, the bandgap of PEDOT: PSS/silk fibroin hydrogels, a dual-network photo-curable conductive hydrogel (CDMA), decreased from 2.36 eV to 1.125 eV when PEDOT: PSS was added, as shown in Figure 1 [21,22]. These lack a conventional semiconductor “band gap” as ion migration, not electron excitation across a gap, controls their conductivity. In some cases, researchers evaluate the energy activation for ion transport instead of a band gap. For example, it was discovered that the activation energy of a natural rubber/NaOH hydrogel electrolyte was about 0.1045 eV [23]. These materials’ band gaps are usually adjusted to increase electrical conductivity for electronic and sensor applications. Carbon nanomaterials or conjugated polymers (such as PEDOT: PSS) dramatically narrow the band gap, producing semiconducting properties. Electronically conductive hydrogels (using PEDOT, graphene) have a predetermined electronic band gap, whereas ionic hydrogels (using NaCl, KCl) depend on ion mobility. Band structure and mechanical toughness may be engineered with multi-network hydrogels [2,5].

Hydrogel semiconductors have several uses, especially in bio-interfacing and enhanced sensing. They combine the mechanical and chemical characteristics of hydrogels with the electrical functions of semiconductors. These applications make use of their special qualities, which include increased bio-interactive functions, excellent conformability, and tissue-level softness. When interfaced with biological tissues, the soft hydrogel semiconductors show tissue-level moduli as low as 81 kPa, enabling great conformability. Applications like the direct adherence of a 10 µm thick hydrogel-SC layer to a tendon replica are made possible by this, showcasing the material’s adaptability to uneven and rough surfaces [24]. Foreign body reactions (FBRs) are lessened by hydrogel-SC thin films’ tissue-level modulus. Research has demonstrated that hydrogel-SC films with certain crosslinker concentrations and moduli lead to decreased collagen densities surrounding implants, similar to the low FBR of PAAc hydrogel. For long-term implanted devices, this feature is essential [25]. The photoelectrochemical behavior of hydrogel semiconductors has improved. Hydrogel-SC films exhibit a steady and persistent faradaic current when exposed to a 625 nm laser, demonstrating effective charge transfer between the semiconductor and the electrolyte. This is mostly explained by the hydrogel’s porous structure, which greatly expands the semiconductor–electrolyte interfaces and makes it possible to use electrons and holes in redox processes more effectively. These materials have important photothermal qualities as well. Hydrogel-SC films may quickly heat up in response to laser light, reaching temperatures as high as 78 °C under 30 s. When compared to pristine polymer semiconductor films, this effective heat buildup leads to better photothermal conversion efficiency. Hydrogel-SC can conformally adhere and produce rapid heating when applied to bio-tissue surfaces covered with biofluids, which makes them appropriate for therapeutic applications like wound healing dressings that combine photothermal treatment with ultrasoft modulus, oxygen permeability, and moisturization [19].

**Figure 2:**
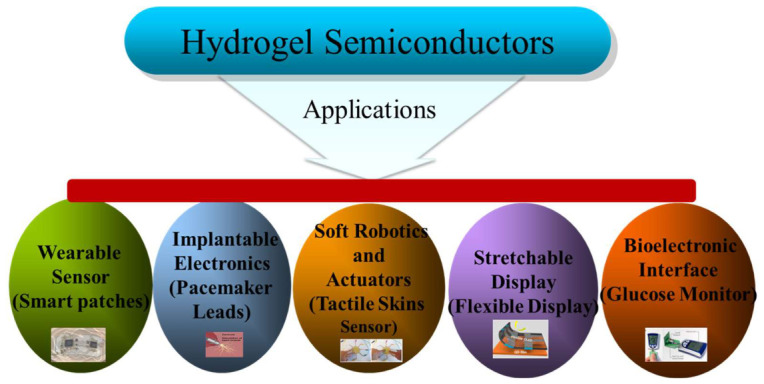
Application field of hydrogel semiconductors.

The mass transport characteristics of hydrogel semiconductors allow volumetric sensing throughout the whole thickness of the semiconducting layer, in contrast to traditional surface-limited biosensing. Higher sensitivity results from more contact sites between bioreceptors and analyte molecules [26]. Large molecules, including macromolecular fluorescent probes (PEG-FITC, 20 kDa), can diffuse more easily across the thickness of hydrogel-SC sheets. The benefit of the hydrogel structure for infiltrating biomolecules is highlighted by the fact that far thinner neat polymer semiconductor films do not exhibit this capacity [27]. Volumetric glucose sensing can be accomplished by crosslinking the hydrogel-SC layer with bioreceptors such as glucose oxidase (GOx). This method shows an efficient way to boost biosensor sensitivity by gradually increasing drain current as glucose concentration rises. Additionally, the glucose sensor has good performance in biological fluids such as sweat and serum, suggesting promise for in vivo sensing applications [28]. For work safety applications, hydrogel semiconductors—more especially, hydrogel-based organic electrochemical transistors, or OECTs—are used as wearable, real-time oxygen gas monitors. In order to prevent injuries and fatalities, the main use of these devices is to monitor oxygen levels in cramped areas. In OECT structures, hydrogels are used in place of liquid electrolytes. This modification is essential for the electrochemical detection of gaseous substances that penetrate and dissolve in the hydrogel, resulting in a wearable sensor that is small and operates in real time. Gaseous oxygen may be converted into an electrical signal due to the hydrogel’s quick oxygen solubilization. The PEDOT: PSS (poly(3,4-ethylenedioxythiophene): poly (styrene sulfonate)) active layer’s electrocatalytic activity aids in this process. The device’s wearability and simplicity of handling are enhanced by the application of a thin hydrogel covering (30 µm). Because of this, it may be incorporated into helmets or worker dungarees as conventional personal protection equipment. The hydrogel-based OECTs show low power consumption (less than 40 µW) and similar sensitivity. Because of this, they may be used for ongoing oxygen monitoring in the crucial 13–21% (*v*/*v*) range for worker safety. These sensors work even when bent because they can be printed on thin plastic surfaces. For wearable applications, this flexibility is crucial because it enables the gadget to adapt to curved surfaces or clothing without sacrificing functionality. Strong and dependable sensing benefits from the OECT architecture’s strong output signal amplification (10^3^–10^4^) and built-in signal filtering [29]. Hydrogel semiconductors can be used in the devices to make them convenient, as shown in Figure 2.

### 2.1. Hydrogel-Based n-Type Semiconductors

A semiconducting material, such as silicon or germanium, that is doped with trace amounts of donor-type atoms is referred to as an n-type semiconductor. The presence of these donor atoms, which possess an additional valence electron compared to the host material, enhances electrical conductivity by facilitating the flow of more free electrons through the crystal lattice when an electric field is applied [30]. Semiconductors are typically composed of group V elements, including phosphorus (P), arsenic (As), and antimony (Sb). These materials are characterized by the presence of free electrons, which contribute to their electrical conductivity. Compared to intrinsic semiconductors, extrinsic semiconductors demonstrate significantly enhanced conductivity due to the increased availability of charge carriers facilitated by doping processes. As a result, semiconductors play a crucial role in modern electronics, serving as the foundational components in diodes, transistors, and integrated circuits. The majority of n-type organic semiconductors are electron-transporting (ET) substances. Their development has historically received less attention than that of p-type materials. There is no one “universal” material that can be used in all organic electronic applications, such as organic field-effect transistors (OFETs), organic light-emitting diodes (OLEDs), or organic photovoltaics (OPVs). This is because the design requirements for ET materials are very application-specific. It is essential to choose the right material and understand how it interacts with other elements in the system. Among the particular n-type organic semiconductor materials are fullerene, acylene diimides, siloles, and others [31]. Although n-type organic semiconductors have seen significant breakthroughs, there are still issues with toxicity, ease of manufacturing, synthesis, purification, operational stability, and device performance. Future work should concentrate on generating more air-stable and solution-processable n-type semiconductors, inventing novel stable dielectric materials, and comprehending the causes of the comparatively poor performance of planar molecules in OPVs [16,32].

**Figure 3:**
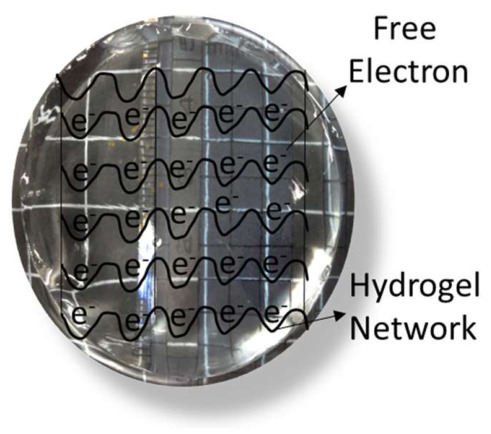
n-type hydrogel semiconductor.

However, a hydrogel network embedded with a water-soluble n-type semiconducting polymer, typically a conjugated polymer featuring electron-donating groups, facilitates electron transport through the hydrated matrix shown in Figure 3. This structure is referred to as an n-type semiconducting hydrogel. The objective of these hydrogels is to enhance high electron mobility, sustain on/off switch behavior, and facilitate the construction of logic circuits and signal amplification. The primary characteristics of the n-type hydrogels include water solubility, multiple network structures, biocompatibility, and signal amplification. In 2024, Ting Lei’s team at Peking University published a study demonstrating the first semiconducting hydrogel logic circuits, which effectively amplified electrophysiological signals in situ and showcased exceptional switching properties where a water-soluble cationic n-type polymer served as the basis for the hydrogels, which formed single and multi-network architectures while maintaining good conductivity and mechanical adaptability [33].

n-Type semiconductor hydrogels are perfect for interacting with biological systems because they are naturally soft, water-rich, and tissue-like. They improve signal quality in biosensing applications by lowering the interfacial impedance between biological tissues and electronics. Semiconducting hydrogels can be processed in water, in contrast to many organic semiconductors that need hazardous organic solvents. As a result, they are safer and more suited to manufacturing procedures and biological habitats. In order to build multi-network systems with certain characteristics like stretchability, adhesion, and responsiveness, semiconductor hydrogels can be mixed with other hydrogel networks. In contrast, organic semiconductors are usually brittle or hard, which restricts their application in dynamic biological environments [34].

The study has developed novel techniques that incorporate water-soluble semiconducting polymers into hydrogel networks to create n-type semiconductor hydrogels. The key materials and components involved in preparing these semiconducting hydrogels are conjugative polymers poly(p-pyridyl vinylene (P(PyV)), particularly developed for water solubility and n-type semiconducting behavior. These polymers are designed to work with hydrogel matrices while retaining electrical conductivity [33]. Polyacrylamide (PAAm) or polyvinyl alcohol (PVA) is a common hydrophilic polymer used to form hydrogel matrices, which provide special properties of hydrogel mentioned earlier. For the crosslinking agent, the N, N′-methylenebisacrylamide (MBAA) or glutaraldehyde is used to form the 3D structure of hydrogel as a crosslinking agent, depending on the base polymer. The tetrahydron (THF), toluene, and dymethylformamide (DMF) are frequently utilized in the synthesis of the precursors for semiconducting polymers in solvent and reaction conditions. To prevent oxidation, reactions are usually conducted in a nitrogen environment.

**Figure 4:**
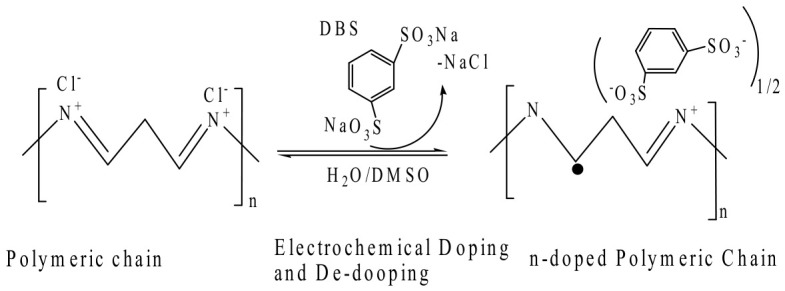
The dianion exchange reaction, the electrochemical doping–dedoping of DBS of ammonium chloride containing polymer chain.

The electron transport dynamics in n-type semiconducting hydrogels, particularly those synthesized from water-soluble polymers like (P(PyV)), represent a fascinating intersection of hydrogel chemistry and the principles of organic semiconductor physics. This integration elucidates the mechanisms by which these materials can effectively facilitate charge transport, thereby enhancing their potential applications in various electronic and optoelectronic devices. The disodium 1,3-benzenedisulfonate (DBS) and other di-anionic crosslinkers engage electrostatically with the cationic polymer to produce hydrogel polymers. As a result, a stable, porous three-dimensional network is produced that maintains ion mobility and water retention without sacrificing electrical conductivity. Anions are released from the matrix during electrochemical doping, which raises the Fermi level above the conduction band minimum (CBM) and creates polaron absorption bands (500–800 nm), which makes electron hopping easier. With an electron mobility (µ) of 0.23 ± 0.01 cm^2^/V. s and a high volumetric capacitance (C) of 485 ± 56.3 F/cm^3^, the hydrogels achieve a benchmark µC value of 112 ± 6 F/cm^−1^·V^−1^·s^−1^, which is among the greatest for n-type organic semiconductors. Figure 4 illustrates the electrochemical doping–dedoping of DBS on a polymeric chain containing tertiary ammonium chloride to produce semiconductor hydrogels. These characteristics make these hydrogels perfect for bioelectronic applications, including ECG, EEG, and ECoG sensing, because they allow for quick switching (on/off timing 1.58/0.18 ms), large on/off ratios (>10^7^), and effective signal amplification in organic electrochemical transistors (OECTs).

The n-type semiconductor hydrogels are perfect for bioelectronic applications because they provide an attractive blend of biocompatibility with excellent electronic performance, offering high volumetric capacitance, low power consumption, fast switching, high gain, mechanical flexibility, and the ability to amplify weak biosignals. While semiconductor hydrogels offer several advantages, they also come with notable limitations that impact their performance and application. The “frequency-dependent performance” of MNH-based devices is a concern, as amplification efficiency drops at higher frequencies due to slower ion migration, with a critical cut-off around 100 Hz. This limits high-speed signal processing applications. Current spray-coating techniques achieve modest “patterning resolutions” of about 200 µm, hindering the integration of high-density circuits essential for advanced electronics. There is also a “trade-off between material rigidity and performance,” where higher concentrations of semiconducting polymers enhance conductivity but reduce stretchability, complicating the balance between flexibility and functionality. The lack of “limited p-type counterparts” restricts the development of fully complementary circuits needed for complex logic operations. Additionally, “environmental stability” is a concern, as these hydrogels remain stable in water but can degrade under prolonged exposure to ionic solutions or mechanical stress without protection. Addressing these interconnected issues is vital for the practical use of semiconductor hydrogels in electronics [33].

### 2.2. Hydrogel-Based p-Type Semiconductors

p-type semiconductors are materials specifically engineered to predominantly conduct positive charge carriers, referred to as holes. These materials are essential for several electronic applications, especially in organic field-effect transistors (OFETs) and other printable and flexible electronics.

p-type organic semiconductors (OSCs) are designed to promote hole mobility. The V-shaped dinaphtho[2,3-b:2′,3′-d]-thiophene (DNT-V) π-core was particularly designed for p-channel organic field-effect transistor (OFET) applications. The design of p-type OSCs frequently requires precise molecular orbital arrangements to maximize effective intermolecular orbital overlaps, stabilize crystal phases, and decrease harmful molecular movements in the solid state. This also seeks to increase solution processability. Bend-shaped molecules, such as DNT-V, are examples of such designs. They have an intrinsic dipole moment that is absent from linear molecules, which helps to stabilize the crystalline phase and increase solubility. One of the most important electrical properties of p-type semiconductors is the ionization potential (IP), which is the energy required to remove an electron and create a hole. Lower IP values are frequently favored for efficient hole injections and reduced threshold voltages in OFET processes. For example, the IP was much lower at 5.21 eV when the π-conjugation was prolonged in DAT-V compared to 5.72 eV in DNT-V. The charge-carrier mobility (μ) of p-type OSCs is frequently used to assess their performance. The mobilities of high-performance OSCs have exceeded 10 cm^2^ V^−1^ s^−1^. One N-shaped OSC achieved a mobility of 16 cm^2^ V^−1^ s^−1^, demonstrating the higher performance of bent-shaped OSCs [35].

**Figure 5:**
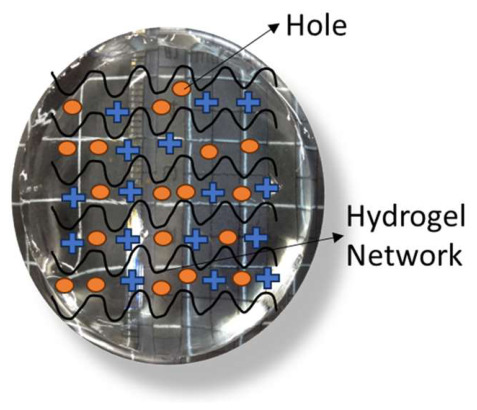
p-type semiconductor.

When exposed to extremely strong light–matter interaction, p-type organic semiconductors, such as regioregular poly(3-hexylthiophene) (rr-P3HT), show intriguing characteristics. Their electrical and optoelectronic properties can be strongly influenced by this connection. The bulk of charge carriers in p-type semiconductors are holes, which are found in the valence band. In contrast, the main carriers of n-type semiconductors are electrons in the conduction band. The delocalized nature of collective states is responsible for the enhanced transport qualities in the strong coupling regime. This indicates that hybrid light–matter states have an impact on the valence band holes in p-type semiconductors. Increased conductivity in the dark state suggests that polaritonic states affect the charge-carrying valence band even in the absence of light. According to the results, strong coupling can enhance optoelectronic characteristics, which makes it pertinent for technological uses like photovoltaics and photodetectors [36]. A p-type semiconducting gel, as used in the study, is a polymeric gel that is intended to contain positive charge carriers, similar to p-type inorganic semiconductors such as silicon and germanium shown in Figure 5. In particular, pyranine (8-hydroxypyrene-1,3,6-trisulfonic acid, trisodium salt) was used to dope the polyacrylamide gel. Na^+^ and SO_3_^−^ ions are the counter ions and side groups, respectively, in this pyranine [37]. The gel’s polymerization is when the doping process takes place. Through radical addition, the pyranine’s hydroxyl (OH) group chemically links to the polymer strands. By forming stable charged sites inside the gel, this binding essentially dopes it with positive counter ions [38]. A number of variables, including the doping agent concentration and swelling ratio, can affect the electrical characteristics of these p-type and n-type gels. Similar to traditional Si and Ge diodes, the work shows that p-type and n-type gels may create a p-n junction that rectifies current when they come into close contact. Doped gels exhibit substantially greater initial current values than undoped (or “neat”) gels at a particular swelling ratio and constant applied voltage. The higher the doping concentration, the higher the net charge in the gel [37]. Polyacrylamide gel is the main polymeric matrix utilized for the p-type semiconducting gel. Acrylamide (AAm), poly (vinyl alcohol) (PVA), poly (ethylene glycol) diacrylate (PEGDA), and N, N′-methylene bisacrylamide (BIS) as the crosslinking agents are used in a free radical crosslinking co-polymerization method to create this gel. The polymerization process is started by ammonium persulfate (APS). Throughout the synthesis process, the ratios of AAm to BIS and AAm to APS are maintained constant for every sample [39,40]. The polyacrylamide gel is doped with trisodium salt, or pyranine, more precisely 8-hydroxypyrene-1,3,6-trisulfonic acid. The presence of SO_3_^−^ ions as side groups and Na^+^ counter ions in this pyranine is essential for its p-type semiconducting characteristics. Through radical addition during polymerization, the pyranine chemically attaches itself to the polymer strands via its hydroxyl (OH) group, forming stable charged sites doped with positive counter ion [37]. A polymeric matrix, such polyacrylamide gel, is doped with certain ionic chemicals, such as pyranine (8-hydroxypyrene-1,3,6-trisulfonic acid, trisodium salt), to produce p-type semiconductor gels. Using its hydroxyl (OH) group and radical addition, the pyranine chemically bonds to the polymer strands during the polymerization process. By creating stable charged sites inside the gel, this chemical binding successfully introduces positive counter ions. The gel’s p-type semiconducting property is due to the existence of these positive counterions. These p-type gels’ electrical characteristics depend on a number of variables, including the doping agents’ concentration and swelling ratio. The gel’s net charge rises in direct proportion to the doping agent’s concentration. At a given swelling ratio and constant applied voltage, the initial current values for doped gels are substantially greater than those for undoped (or “neat”) gels when an external voltage is applied. Ionic conductivity, in which the charge carriers are ions in the gel’s water phase, is the main cause of the current in these gels. The current density decreases as a result of these charge carriers building up on the electrodes over time.

A p-n junction is created when p-type gels and n-type gels come into close contact. Like traditional silicon (Si) and germanium (Ge) diodes, this junction exhibits current rectification. The doping effect is the primary cause of the one-way flow of current at the interface between the cationic (p-type) and anionic (n-type) gels. In order to conduct electricity and create rectifying junctions when paired with n-type gels, p-type semiconductor gels work by introducing stable positive charge carriers by chemical doping [37,41]. To create complementary inverters, n-type semiconducting hydrogels are combined with p-type semiconductor gels, more precisely, P(lgDPP-MeOT_2_). For bioelectronic devices to process and amplify signals, these inverters are essential. p-Type and n-type materials can be combined to create circuits with great performance, as evidenced by a high gain value (V_out_/V_in_) of −1 [13,42].

### 2.3. Factors Influencing Semiconductor Properties of Hydrogel-SC

Long-term stability and whether a hydrogel behaves as an ionic conductor, a mixed ionic–electronic conductor, or in a true semiconducting phase under physiological conditions (e.g., pH 7.4, ionic strength ~0.15 M) are determined by solvent composition, co-solvents, electrolyte identity/concentration, and pH. These factors also strongly control hydrogel swelling, ionic vs. electronic charge transport, dopant stability, and interfacial electrochemistry [43]. By altering ion dissociation, dielectric screening, viscosity (ion mobility), polymer–solvent interactions, and microstructure, the choice of solvent significantly influences a hydrogel’s semiconductor behavior. For bioelectronic or soft devices, labs give priority to solvent dielectric constant and viscosity to adjust conductivity and stability [44]. In contrast, co-solvents that lower water activity (glycerol, Deep Eutectic Solvent (DES)) maintain electronic percolation and dopant stability. Water uptake increases interchain spacing and the local dielectric constant, favoring ion transport and decreasing electronic overlap between conjugated segments [43,45]. Phase separation, crosslinking coordination, and polymer swelling are all altered by solvent polarity and hydrogen bonding, which also modify charge transport percolation pathways [46].

The main design parameters for sensors, wearable electronics, and energy devices are the choice of ion type, concentration, and solvent. Electrolytes may regulate hydrogel semiconductor performance by setting ionic conductivity, modulating electronic carrier density and interfacial charge transfer, and altering mechanical swelling/adhesion. Ionic conductivity (mS·cm^−1^ scale), interfacial impedance, and swelling-driven mechanical changes are important observable factors that impact device stability, speed, and biocompatibility. In typical hydrogels, mobile ions serve as the primary conduction conduit; conductivity is increased by increasing salt concentrations and tiny, highly mobile ions [47]. Ions screen charges, change the Fermi level, and can function as electrochemical dopants in semiconducting hydrogels (such as water-soluble n-type polymers), changing the carrier density, mobility, and on/off ratio [33]. Electrolytes facilitate capacitive or faradaic coupling and lower electrode–hydrogel impedance; the anion/cation selection influences redox-side reactions and double-layer development [48].

The ionization state of polymer functional groups, which modifies charge carrier density, swelling (pore size), and ion mobility, is the main way that pH affects hydrogel semiconductor conductivity. In general, acidic conditions reduce ionic conductivity, while basic conditions increase; the amount varies depending on polymer chemistry, dopants (such as conductive fillers), and ionic strength [49]. The charge state of polymers containing basic (–NH_2_) or acidic (–COOH) groups changes with pH: protonation neutralizes groups and decreases mobile charge, whereas deprotonation results in fixed charges and mobile counterions that boost ionic conductivity [50]. Reduced ionization results in deswelling and fewer conductive routes; increased ionization increases osmotic pressure inside the network, leading to swelling, bigger pore channels, and greater ion mobility. By changing filler dispersion, polymer doping level, or interfacial charge transfer, pH can modify the percolation network in hydrogels containing conductive polymers or nanofillers (such as PEDOT:PSS, graphene oxide), regulating both electrical and ionic conductivity [43,51].

### 2.4. Application and Working Mechanism of Hydrogel-SC

Hydrogel-based semiconductors are enabling the development of soft, biocompatible electronics—from implantable amplifiers and wearable sensors to thermoelectric generator (TEG) soft transistors and logic circuits—by combining tissue-like mechanics with true electronic charge transport; recent work demonstrates n-type semiconducting hydrogels that make complementary circuits and high-gain bioamplifiers possible [44,52]. Table 1 lists the hydrogel-based n-type and p-type semiconductors applicable in different fields of study and performance. Jiafu et al. prepared both n-type and p-type semiconductor hydrogels for thermoelectric cells applicable for low-grade heat harvesting. Particularly in ion thermoelectric cells (i-TECs), n-type and p-type hydrogel semiconductors function via a temperature-sensitive phase transition that affects the redox ion concentration differential and produces a Seebeck effect. The triiodide/iodide (I_3_^−^/I^−^) redox couple, which is created by submerging the hydrogel in an I_3_^−^/I^−^ solution, is the main mechanism used by these hydrogel i-TECs. Because of its lower charge density, the triiodide species (I_3_^−^) is more hydrophobic than iodide (I^−^), making it more vulnerable to hydrophobic interactions.

**Figure 6:**
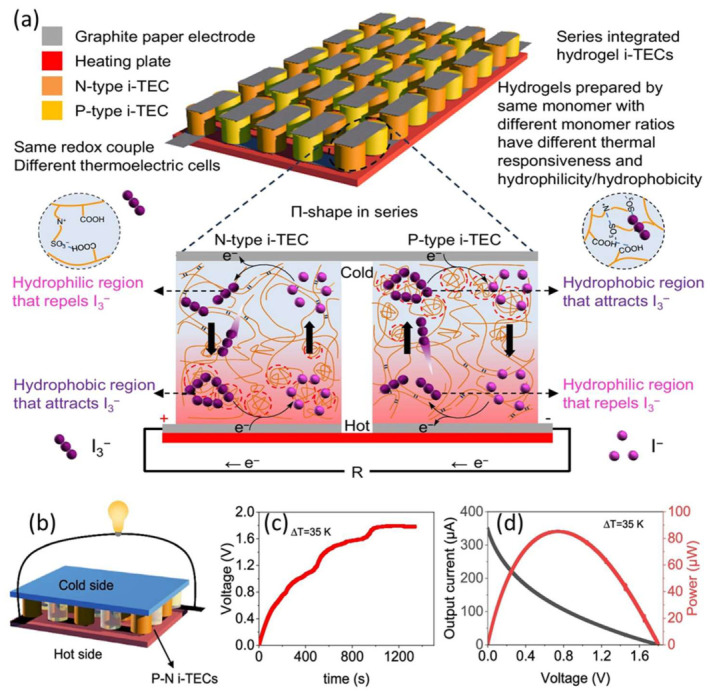
(**a**) The n-type and p-type iTECs are sandwiched between two flexible substrates and coupled alternatively in a Π-shape using graphite paper electrode. (**b**) Integration of series. (**c**) Ten pairs of n-p i-TECs’ output voltage. (**d**) Ten pairs of n-p i-TECs’ power and current output. (Reused from the reference [53].).

The temperature-sensitive hydrogel undergoes a phase shift as part of the fundamental process. As seen in Figure 6a, the p-type i-TEC becomes hydrophilic, and the n-type i-TEC becomes hydrophobic when the temperature rises over its phase transition threshold. The hot side of an n-type i-TEC turns hydrophobic, attracting triiodide (I_3_^−^), whereas the cold side turns hydrophilic, repelling I_3_^−^. As a result, I_3_^−^ concentrations are greater on the hot side and lower on the cold side. Electrons move from cold to hot via the external circuit during the oxidation process (3I^−^ − 2e^−^ → I_3_^−^) at the cold side and the reduction reaction (I_3_^−^ + 2e^−^ → 3I^−^) at the hot side. In contrast, the cold side of a p-type i-TEC becomes hydrophobic and attracts I_3_^−^, while the hot side becomes hydrophilic and repels I_3_^−^. As a result, there are more I_3_^−^ on the cold side. Compared to the n-type i-TEC, the oxidation and reduction processes are reversed. The hydrophobic areas of the hydrogel and triiodide interact strongly to increase the concentration difference of I_3_^−^, which raises the Seebeck coefficient. For example, with a temperature differential of 15 K, n-type i-TECs reach a Seebeck coefficient of 7.7 mV K^−1^, whereas p-type i-TECs obtain −6.3 mV K^−1^. As seen in Figure 6b, a larger voltage and output power may be obtained by connecting many n-type and p-type i-TECs in series. For instance, with a temperature differential of 35 K, ten pairs of n-type and p-type i-TECs linked in series may provide 1.8 V and 85 µW (Figure 6c,d). Compared to previously published triiodide/iodide-based i-TECs, this performance is better [53].

**Figure 7:**
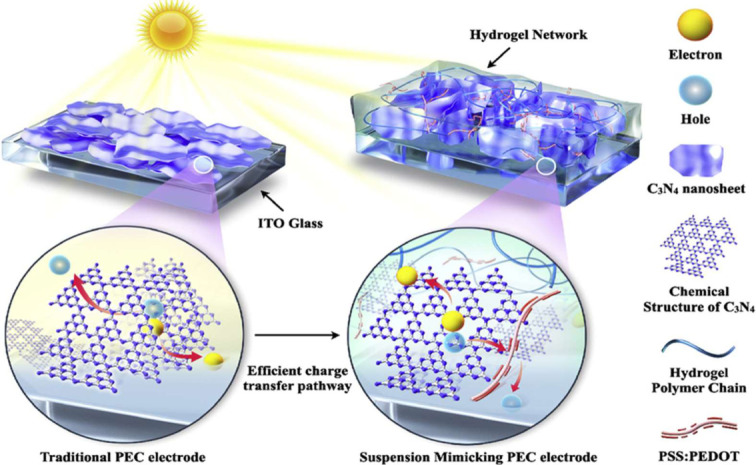
A hydrogel-based film made of B-g-C_3_N_4_ nanosheets with charge transfer routes and a conventional carbon nitride layer that mimics suspension. (Reused from reference [54].).

**Figure 8:**
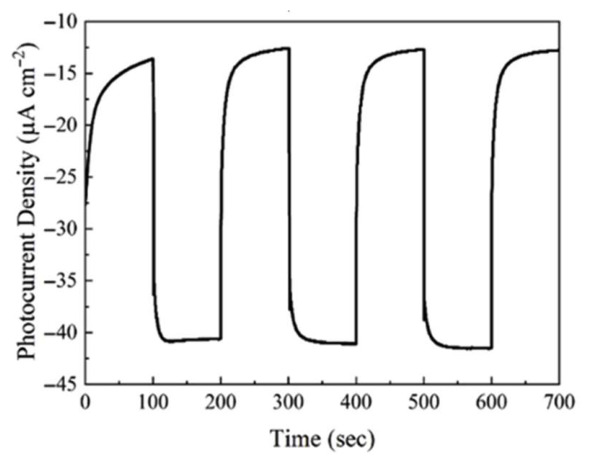
Photocurrent density-versus-time plots of the flexible B-g-C_3_N_4_ hydrogel photocathode measured at 0.3 V potential versus RHE. (Reused from reference [54]).

Wenwen et al. designed the hydrogel-based n-type polymer photocathode to effectively collect electrons for solar-driven water splitting, namely the hydrogen evolution shown in Figure 7. It uses boron-doped graphitic carbon nitride (B-g-C_3_N_4_) nanosheets inside a hydrogel matrix. Several important steps are involved in its operation:

The hydrogel matrix is essential because it gives the B-g-C_3_N_4_ nanosheets a suspension-like environment. Similar to a suspension system, this keeps the nanosheets from aggregating and permits the free flow of gas and water molecules. This configuration is intended to get around the drawbacks of conventional photoelectrochemical (PEC) films, which frequently have inadequate efficiency because the benefits of suspension systems are lost. The B-g-C_3_N_4_ nanosheets produce electron–hole pairs when they are exposed to sunlight because they absorb photons. For photocatalytic activities, boron doping increases visible light absorption and helps close the band gap. In order to create shallow surface trap states, boron atoms were purposefully doped into the graphitic carbon nitride. In order to effectively capture electrons and separate charges, these trap states are essential for collecting photogenerated electrons. Despite the semiconductor’s n-type nature, this technique is essential for producing cathodic photocurrent. Upward band bending can prevent electrons from building up on the surface of n-type semiconductors. Nevertheless, this band bending is lessened by the hydrogel’s nanosheet structure, which makes it easier for electrons to gather on the surface for reduction processes. Additionally, by reducing the charge migration distance, the nanostructure improves charge transfer efficiency and lowers charge recombination. After being effectively moved to the electrolyte, the electrons trapped by the shallow-level boron defects take part in the water reduction process to create hydrogen. The effective charge transfer kinetics at the semiconductor–electrolyte interface facilitate this process. Charge separation is further aided by the attachment of a hole conductor, such as PEDOT: PSS, to the B-g-C_3_N_4_ surface, which offers an alternate route for effective hole conveyance. As seen in Figure 8, a typical hydrogel-supported, boron-doped, n-type graphitic carbon nitride nanosheet photocathode attained a current density of −30 µA/cm^2^ at 0.3 V vs. reversible hydrogen electrode (RHE), an order of magnitude greater than a normal carbon nitride photoelectrode (−3 μA/cm^2^). The maximum photocurrent was produced at the ideal boron level of 4.99% [54].

**Table 1:** Hydrogel-based semiconductors.

S.N.	Hydrogel Semiconductor	Types of Semiconductors	Field of Application	Performance	Reference
**1**	(a) Polyacrylamide gel doped with pyranine (8-hydroxypyrene-1,3,6-trisulfonic acid, trisodium salt) (b) N-isopropylacrylamide (NIPA) gel doped with methacrylamidopropyltrimethyl ammonium chloride (MAPTAC)	(a) p-type (b) n-type	Flexible and biocompatible electronics	Electrical behavior is precisely controllable through their chemical composition and physical state.	[37]
**2**	(a) Polyacrylamide gel doped with pyranine (b) Polyacrylamide gel doped with Tetraallil ammonium bromide	(a) p-type (b) n-type	Flexible and biocompatible electronic circuits	Significant rectification is observed in junctions formed with doped p- and n-type gels	[55]
**3**	Disodium 1,3-benzenedisulfonate doped with poly (p-pyridyl vinylene (P(PyV))	n-type	Bioelectronics	(a) Electron mobilities values up to 120 F cm^−1^ V^−1^ s^−1^ (b) The maximal volumetric capacitance (C) is 485 F cm^−3^ and electron mobility (μ) is 0.25 cm^2^ V^−1^ s^−1^	[33]
**4**	Methacrylic acid (MAA), 3-dimethyl(methacryloyloxyethyl) ammonium propanesulfonate (DMAPS) and I_3_^−^/I^−^ Redox Couple	(a) p-type (b) n-type	Ionic thermoelectric cells	A voltage of 1.8 V and an output power of 85 µW at temperature difference of 15 K	[53]
**5**	Boron-doped graphitic carbon nitride (B-g-C_3_N_4_) nanosheet and (PEDOT: PSS)	n-type	Solar-driven water splitting (hydrogen evolution)	A current density of −30 µA/cm^2^ at 0.3 V, Internal Photon to Current Conversion Efficiency (IPCE) of 1.04% at 360 nm.	[54]
**6**	PEDOT: PSS/PVA/CuCl_2_	n-type	Thermoelectric generator	Outstanding thermopower (α) of −33.8 mV/K,	[56]

## 3. Scope of Improvement and Future Prospect

The total conductivity is still lower than that of conventional semiconductors, even when semiconducting polymers can be incorporated. Their performance in high-speed or high-power electrical applications is thus constrained. Integration is challenging because stiff components and soft hydrogels have different mechanical characteristics. Interface failure or delamination may result from this. It is difficult to achieve homogeneous dispersion of semiconducting elements in the hydrogel matrix, which demands tight control over the crosslinking and synthesis procedures.

One important development is the creation of n-type semiconducting hydrogels. These are particularly difficult to synthesize since n-type polymers are easily oxidized in air or water, especially in aquatic settings. Nevertheless, if these materials are successfully fabricated, bioelectronic signals can be actively amplified, creating new bioelectronics opportunities. The performance of current p-type and n-type semiconducting hydrogels is known to differ, despite the promising performance of certain p-type hydrogels such as P(lgDPP-MeOT_2_). One major obstacle is still the creation of p-type semiconducting hydrogels that can perform as well as their n-type counterparts. To guarantee the best possible signal conversion and amplification efficiency in hydrogel-based flexible amplifiers and to avoid problems like reduced device lifespan and signal quality degradation, similar electrical performance is necessary. This gap has to be filled in order to enhance the capabilities of p-type semiconductor gels. To address the shortcomings of current semiconductor hydrogels, innovative designs can be introduced in following research. The integration of nanomaterials such as graphene, MXenes, or carbon nanotubes may improve conductivity and mechanical strength. Composite hydrogels with dual networks (e.g., tough polymer + conductive filler) show potential. For wearable electronics and soft robotics, hydrogels with reversible bonds or dynamic crosslinking can be designed to be stretchable and self-repairing.

By creating percolating conductive networks and acting as high-aspect-ratio load-bearing fillers that interact strongly with the polymer matrix (via π–π, hydrogen bonding, ionic or covalent linkages), graphene or its derivatives (graphene oxide (GO), reduced graphene oxide (rGO)) may improve mechanical strength and electrical conductivity in semiconductor hydrogels. These effects are frequently optimized by adjusting graphene loading, dispersion, and interfacial chemistry, and they have been extensively documented in recent reviews and experimental investigations [57,58]. By creating continuous, highly conductive 2D networks that facilitate rapid electron transport, enhance interfacial charge transfer with the polymer matrix, provide mixed ionic–electronic conduction, and improve mechanical stability and freeze/oxidation resistance, MXene addition significantly increases hydrogel semiconductor conductivity [59,60]. By creating a percolating, high-mobility carbon network inside the soft polymer matrix that permits electronic charge transport (metallic/semiconducting CNTs), enhances carrier mobility, and produces mixed ionic–electronic conduction, carbon nanotubes (CNTs) increase hydrogel semiconductor conductivity. This makes CNT-filled hydrogels much more conductive and beneficial for flexible electronics and sensors [61,62]. The addition of biomolecules or responsive components can enhance biocompatibility and facilitate intelligent sensing, making them perfect for tissue engineering and medical diagnostics. The ability to precisely manipulate structure and function through techniques like 3D printing, microfluidics, and photopatterning will enable the creation of multipurpose platforms and unique device architectures.

## 4. Conclusions

Hydrogel-based semiconductors represent a promising yet nascent area of research, where the interplay of soft polymer matrices and electronic functionality offers unique opportunities for innovation. Current systems, primarily based on polyacrylamide and poly(vinyl alcohol), demonstrate the feasibility of integrating semiconducting behavior into hydrogels, but their conductivity and mechanical robustness remain limited. The use of dopants such as disodium 1,3-benzenedisulfonate for n-type semiconductors and pyranine molecules for p-type semiconductors highlights the potential for tailoring charge transport through molecular engineering, though performance is still far from optimal. These challenges underscore the need for deeper exploration into novel raw materials, advanced doping strategies, and hybrid designs that can enhance both electronic and mechanical properties. As the field is still in its early stages, there remains vast potential for breakthroughs—ranging from bio-integrated electronics and flexible sensors to energy devices—where hydrogel semiconductors could play a transformative role. Continued interdisciplinary research will be essential to unlocking their full potential and establish them as viable candidates in next-generation electronic technologies. Throughout this review, we have presented the fundamental knowledge of hydrogel-based semiconductors, encompassing their raw materials, structural properties, and functional mechanisms. This foundational understanding not only clarifies the current state of research but also provides a valuable platform for readers to explore new directions in this emerging field. Ultimately, the insights summarized here may serve as a springboard for immense research opportunities, guiding the development of next-generation hydrogel-based electronic devices with enhanced conductivity, mechanical stability, and multifunctionality.

## Data Availability

No new data were created or analyzed in this study.
